# Supplementary material for: MetaQTL: a package of new computational methods for the meta-analysis of QTL mapping experiments
Source: BMC Bioinformatics. 2007 Feb 8;8:49. doi: 10.1186/1471-2105-8-49 (PMC1808479; doi:10.1186/1471-2105-8-49)
Supplement: Additional File 7 — MetaQTL Package : jar file and tutorial. This Zip archive contains both the MetaQTL JAR file and the files of the tutorial. [file 1471-2105-8-49-S7.zip › org.inra.metaqtl/doc/org/metaqtl/main/package-frame.html]

org.metaqtl.main


org.metaqtl.main

Classes 
  
A2Xml
  
CmdLineParser
  
CmdLineParser.Option
  
CmdLineParser.Option.BooleanOption
  
CmdLineParser.Option.DoubleOption
  
CmdLineParser.Option.IntegerOption
  
CmdLineParser.Option.LongOption
  
CmdLineParser.Option.StringOption
  
ConsMap
  
InfoMap
  
MapView
  
MetaDB
  
MetaMain
  
MMapView
  
MQTLView
  
QTLClust
  
QTLClustInfo
  
QTLModel
  
QTLProj
  
QTLTree
  
Xml2A

Exceptions 
  
CmdLineParser.IllegalOptionValueException
  
CmdLineParser.NotFlagException
  
CmdLineParser.OptionException
  
CmdLineParser.UnknownOptionException
  
CmdLineParser.UnknownSuboptionException
